# New Insights into Polychaete Traces and Fecal Pellets: Another Complex Ichnotaxon?

**DOI:** 10.1371/journal.pone.0139933

**Published:** 2015-10-06

**Authors:** Kantimati G. Kulkarni, Rajani Panchang

**Affiliations:** Biodiversity and Palaeobiology Group, Agharkar Research Institute (MACS-ARI), Pune, Maharashtra, India; Naturhistoriska riksmuseet, SWEDEN

## Abstract

Neoichnological observations help refine paleoichnological records. The present study reports extensive observations on the distribution, morphology, occurrence and association of burrows and fecal pellets of the polychaete *Nereis diversicolor* in the Kundalika Estuary on the west coast of India. Our holistic study of these modern-day traces suggests it to be a complex trace arising from domichnial, fodinichnial and possibly pascichnial behavior of polychaetes. The study for the first time reports extensive fecal pellet production, distribution and their preservation as thick stacks in modern estuarine environment. These observations testify the fossilization potential of pellets and provide an explanation to their origin in the geological record. Their occurrence as strings associated with mounds not only suggests pascichnial behaviour of polychaetes but also allows the assignment of post-Paleozoic *Tomaculum* to the activity of polychaete worms. The production of fecal pellets in such large quantities plays a major role in increasing the average grain size of the substrate of these estuarine tidal flats, thereby improving aeration within the substrate.

## Introduction

Fecal pellets comprise a vital group of trace fossils, especially when found in conditions where preservation of soft bodied organisms is absent or poor. They have been used to reconstruct almost all types of paleoenvironments and paleoecologies throughout time [1–4]. However, systematic neoichnological studies have provided modern analogues to calibrate interpretations of the fossil record. This has led to a better understanding of organismal media interactions, their behavior and preservation potential into the geological record. The present study describes the occurrence, distribution and architecture of a complex trace of polychaetes, giving new insights to pellet-burrow associations reported from the fossil record.

**Fig 1:**
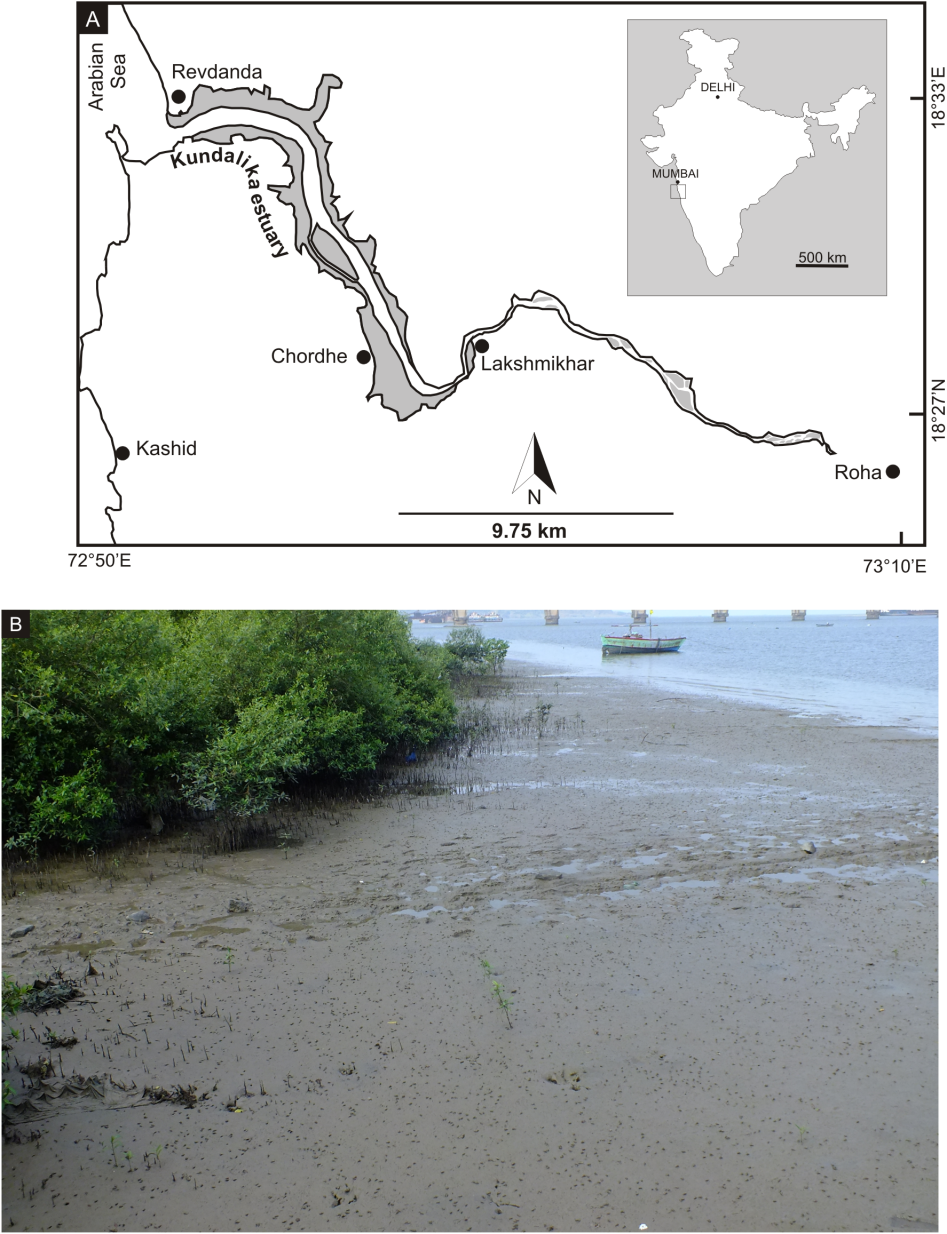
Map of the study area; A. Location of the Kundalika estuary along the west coast of India. The regions shaded in grey denote the tidal flats. Polychaete traces were studied at Revdanda, Chordhe and Lakshmikhar B. Field photograph of the tidal flat at Revdanda.

Polychaete burrows associated with mounds of pellets are the most common traces visible on the vast tidal flats of the Kundalika Estuary. The Kundalika is a major river meeting the Arabian Sea at Revdanda in the Central West Coast of India (Fig 1A), which originates at an altitude of 820 m above sea level about 150 km southeast of Mumbai. It flows in a southeast–northwest direction, has a funnel shaped mouth and the estuary is dominated by semi-diurnal tides. Of the total 40 km length of the estuary, the lower 27 km are the tidal stretch. The width of the channel increases considerably from the upper (150 m) to the lower reaches (600 to 700 m). Upstream the estuary is a drowned valley that opens up into a wide channel with expansive tidal flats in its middle reaches. The tidal flats in the upper estuary (50 to 150 m wide) are characterized by marshlands, whereas those in the middle and lower estuary (100 to 900 m wide) support dense mangrove vegetation (Fig 1B). The study area experiences tropical warm, humid climate throughout the year. The temperature ranges between 25°C and 35°C and the average annual rainfall is 3750 mm.

## Methodology

In order to study the ichnoactivity in the Kundalika estuary, traverses were taken through the tidal flats, in the lower, middle and upper reaches of the estuary. Variation in the distribution of polychaete burrows was observed and recorded in field. The polychaetes which created the burrows were isolated wherever possible and narcotized using pure Ethanol for identification. Pellets associated with the burrows were collected for detailed microscopic observation. Sediment samples were collected in order to determine sediment textures supporting the burrows. Thermo Scientific Orion Star A329 portable, multiparameter meter was used to record environmental parameters such as temperature, salinity, pH and dissolved oxygen (Table 1) on water samples collected at the same sites where shallow cores were extracted for the study.

**Table 1:** Environmental parameters measured on intertidal waters of the estuary.

Site of Sampling	Temperature (°C)	Salinity (ppt)	pH	Dissolved Oxygen (mg/l)
**Lower reaches**	35–36	24.3–25.75	8.03	5–9
**Upper reaches**	34–36	5	6.7	2–4

**Fig 2:**
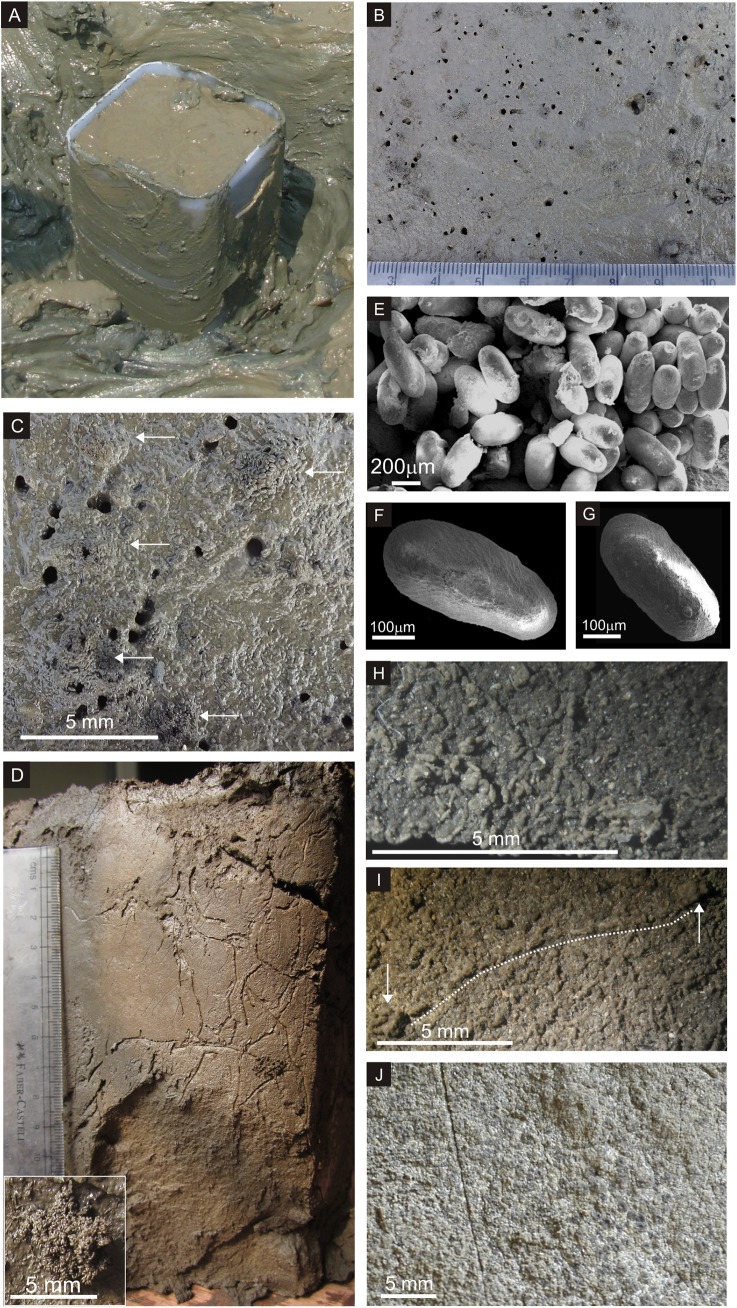
A. The shallow box core being extracted at Revdanda tidal flat. B & C. Field photographs of polychaete burrows associated with pellet mounds. The white arrows in C indicate pellet mounds. Innumerable pellets lie dispersed in the background on the surface of the mudflats. D. Wavy, sinuous and branched burrows of uniform width. Inset: pellet cluster. E. SEM photograph of a pellet aggregate. F & G. SEM images showing surficial striae on pellets. The pellet in F shows an attachment scar in negative relief. H. The undisturbed surface of the box core covered with randomly oriented pellets. Numerous cross-sections of pellets directed towards the reader show dark, homogenous internal structure of pellets. I. One of the several strings of fecal pellets connecting two pellet mounds. J. Lateral surface of box core showing stacked laminae of pellets.

Undisturbed sediment cores, both circular (length 1 m; diameter 11.5 cm) and box-shaped (20 x 18 x 22 cm^3^) (Fig 2A), were extracted from the tidal flats for laboratory studies. The cores were cut open after a week or two, after little desiccation, to enable observations. The dried sediment cores and pellets were observed under the microscope. Scanning Electron Microscopic (SEM) imaging of pellets was done on a Zeiss EVO MA 15 machine in order to describe their surface ultrastructure.

In order to determine the grain size distribution of the substrate, ~15g of sediments collected in three replicates from different locations studied in the estuary were dried overnight at 60°C. Each dried sample was weighed and soaked in distilled water. They were subsequently treated with 10 ml of 10% sodium hexa-metaphosphate to dissociate the clay particles followed by 5 ml of 10% hydrogen peroxide to oxidise the organic matter, if present. The treated samples were wet sieved through 63 μm (250 mesh) size sieve. The sand residue retained over the sieve was dried at 60°C to get the weight of the sand fraction. The filtrate collected in a measuring cylinder was used for pipette analysis to determine the silt and clay fraction in each sample.

It is declared that the field area was in public domain and was not part of any protected area / sanctuary, nor was it any private property. So no permissions were required to sample the study area. Polychaetes are not protected/endangered/scheduled animals.

## Observations

Circular openings of polychaete burrows, visible to the naked eye, dominate the tidal flats in the lower reaches of the estuary. They become more conspicuous in occurrence as well as size, away from the low tide line (~40 m from LTL), as the substrate becomes firm. They are often associated with dispersed fecal pellets (difficult to identify and associate in the field) or with aggregated pellet mounds, located very close to the burrow openings (Fig 2B and 2C). The sediment texture is silty-mud (78% Mud = Silt 48% + Clay 30%). The consistency of the substrate is also controlled by 40–50% water content, causing the pellet mounds to splay. In the vegetated part of the tidal flat the water content of the sediment is low and the ground is comparatively firm.

The burrow openings are well defined, circular in surface manifestation, and vary between 0.8 to 1.2 mm in diameter. The polychaete isolated from the tubular burrows is identified as *Nereis diversicolor*. As observed in field and from sediment cores, the worm makes tubular, branched burrows of uniform width throughout their length. These burrows are associated with a light halo throughout their length. These straight to slightly sinuous burrows extend to a depth of about 20 cm. (Fig 2D). They may or may not be associated with pellet aggregates that reach a maximum size of about 3 mm (Fig 2E). Depending upon their overall size, the pellet aggregates consist of 25 to 1000 pellets each. The individual pellets are compact, well defined and elliptical in shape, with blunt ends. Their long axes range in length from 310 to 450 μm and their short axes from 130 to 220 μm. The length/width ratio (L/W) ranges from 2 to 2.5 (Avg. = 2.3±0.20). The surface of the pellets show inclined, fine striations bifurcating away from the long axes, which could be attributed to their ejection through the anus. (Fig 2F and 2G). The attachment scar along which pellets adhere to one another, is manifested as a broad, shallow furrow (Fig 2F). However, they do not exhibit any differentiated internal structures. The pellets commonly show surface scars and minimal deformation due to compaction.

**Fig 3:**
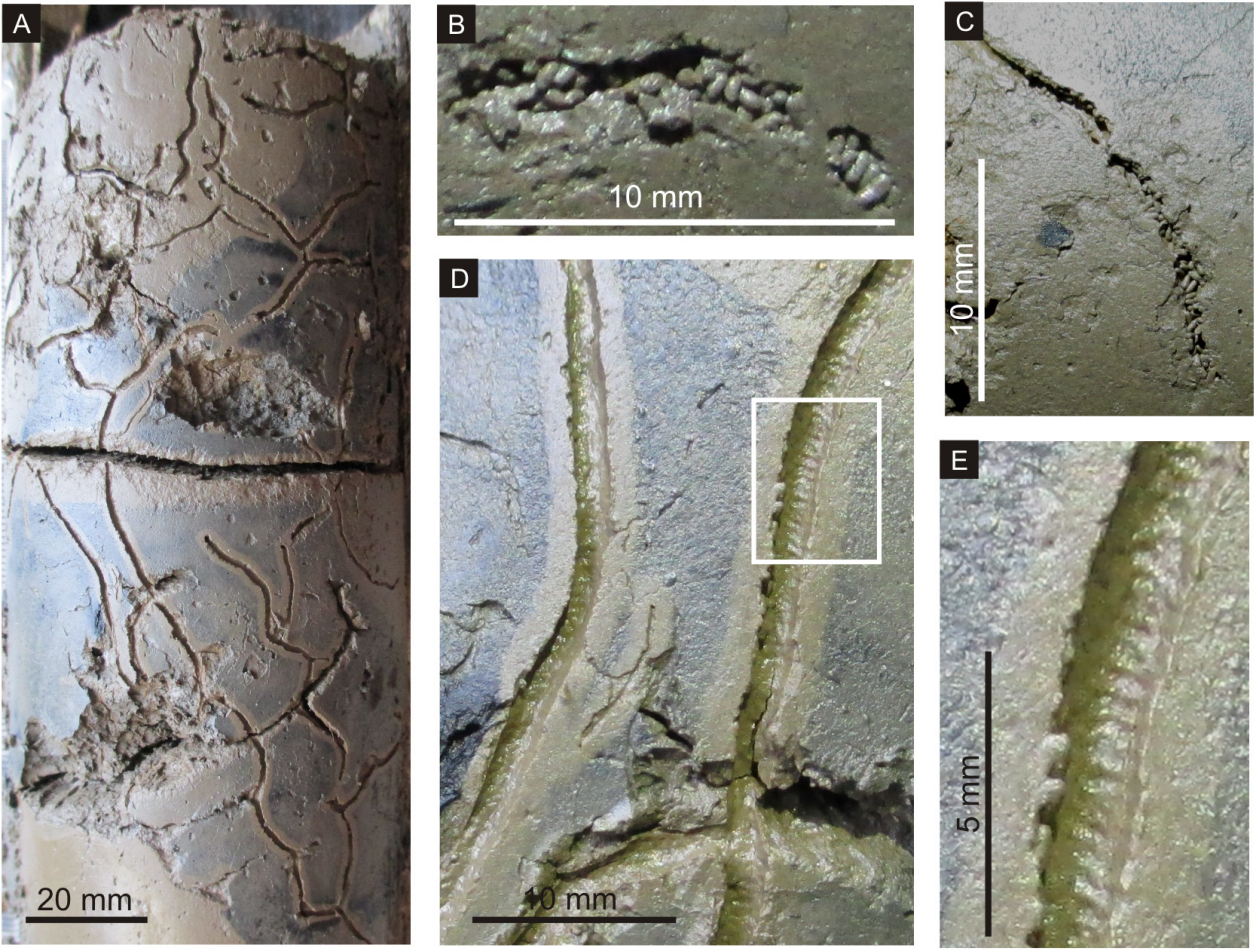
A. Periphery of circular sediment core covered with network of burrows, showing a distinct light colored halo. B & C. Most of the burrows stuffed with randomly oriented pellets. D & E. Many of the burrows show annulations on their walls.

Microscopic observation of the box core surface revealed that the entire surface of the sediment core is covered with a continuous layer of randomly oriented pellets (Fig 2H). The pellets occur as small mounds only when associated with a burrow opening. They also sometimes occur as strings across the surface. These strings commonly connect burrows openings (Fig 2I). Close observation of the side walls of the box cores shows compact layering of pellets, with their circular transverse sections stacked tightly upon each other. The entire surface constituting the lateral side of a core is stacked with pellets bound together (Fig 2J). Deformation of the pellets due to compaction is not observed. The internal structure of the pellets is identical to the texture of the surrounding sediments. The dense accumulation of pellets seems to alter the original texture of the sediments on the tidal flat, from fine grained muddy, to coarser pelleted sands. Examination of the internal, desiccation parting surfaces of the core also reveals randomly oriented, but laminate stacking of pellets, parallel to the tidal flat surface. Upon drying, each of these pellets shows a dark colored core with an external, ultrafine, light colored, shiny lining comprised of crystalline, saline precipitates (Fig 2E). Polychaete burrows are not evident on the surface of the intertidal mudflats in the middle (12 km inland) and upper reaches (24 km inland) of the Kundalika estuary. In the upper reaches, the sediment texture is more muddy (Clay 74% + Silt 25% + Sand 1%) than that in the lower reaches. The flats, though exposed during low tide, are characterized by dense marsh and are always water logged. However, abundant polychaete burrows were observed in the sediment cores obtained at these locations. Drying or loss of water content in the sediments, post collection, could have enabled burrowing and / or these observations. The top surface of the sediment core showed a network of burrows, parallel to the bedding plane. Similar networks of burrows were observed along the periphery of the sediment cores, perpendicular to the sediment surface, up to a depth of about 16 cm (Fig 3A). Most of the burrows were stuffed with ellipsoidal fecal pellets, which showed no preferred orientation (Fig 3B and 3C). At some places, the inner surface of the burrow showed well-defined annulations (Fig 3D and 3E).

The polychaete burrows either occur as circular openings on the tidal flat surface, or as a network of unlined, tubular and irregularly branching burrows. The internal surfaces of these burrows also occasionally display annulations. Very commonly these burrow openings are associated with pellet mounds / aggregates. These burrows are also seen packed with pellets, where the clay percentage and water-logging are considerably high. These pellets also occur as strings across the surface of the tidal flat, which in itself is covered with a blanket of fecal pellets. As seen in Fig 2H the strings often connect two pellet mounds associated with a burrow. Stacking of numerous laminae constituted by pellets, characterize the cross section of the tidal flat in the lower reaches of the estuary.

## Discussion

Polychaete burrows associated with pellet mounds have been widely studied and reported from modern settings [5–8]. However, the present study for the first time reports them as a composite trace. The observations compiled above provide a holistic view of the intense bioturbation by polychaetes and have ichnological, sedimentological and paleoenvironmental implications.

### Ichnological implications

The individual components constituting this complex trace, when seen in isolation, can be referred to various ichnotaxa / fossil analogues. The burrows can well be compared with the ichnogenus *Trichichnus*, which is reported to be eurybathic, associated with fine grained sediments, and attributed to marine meiofaunal deposit-feeders [9]. However, the same burrow featuring annulations can be compared with *Planolites annularius* [10]. Burrows filled with pellets are co-relatable with the ichnogenus *Alcyonidiopsis* [10] because this ichnogenus and all its species are characterized by burrows with a diameter of 5 to 7 mm and pellets with diameters of 400 to 600 μm, which are much larger than those observed in the present study. The pellets in the present study and their aggregates are most comparable with *Tibikoia* [11], though again differ by being much smaller in size. *Tibikoia* is an ichnogenus used to describe oblong shaped fecal pellet aggregates only. They have been attributed to polychaetes and are about 1 mm in length and 0.5 mm in diameter; individual aggregates attain maximum diameter of 20 mm. *Tibikoia* is now regarded as the junior synonym of *Coprulus* [12]. Baluk and Radwanski[13] described a new ichnospecies *Tibikoia santacrusensis* and attributed the pellets to polychaete annelids, presumably related closely to the present-day species *Heteromastus filiformis* (Claparède). The burrows of *H*. *filiformis* are also single aperture burrows showing subsurface branching identical to those found in the present study and are also reported from estuarine mud areas [14–15]. The strings of pellets, interrupted by small mounds or aggregates seen on the surface of the tidal flat, can be compared with the ichnogenus *Tomaculum* [16]. This ichnogenus describes strings of elliptical fecal pellets up to 10 cm long and 1 to 2 cm wide and lying on the bedding plane. Pellets therein (1–5 mm in length and 0.5 to 1.5 mm in diameter) occur in clusters which are loosely strung together and are attributed to trilobites or annelid worms [17]. However, fossil fecal pellet strings identifiable as *Tomaculum* in post-Paleozoic rocks cannot be attributed to trilobites. In contrast to our existing knowledge of organisms creating such pellets, post-Paleozoic *Tomaculum* can now also be attributed to the activity of polychaete worms, based on the observations of the present study. Additionally, the fact that the pellet strings recorded on the surface of the tidal flats, often connect two pellet mounds associated with a burrow, is suggestive of the pascichnial behavior of the polychaetes.

### Sedimentological implications

Although beds of pelleted muds have not been reported from modern environments, early diagenetic, syndepositional pellet sands retaining their original texture have been reported from Danish Tertiary sediments ([3] and references therein). In that study, Friis suggested the fast deposition of a 10 cm layer of pelleted mud in an environment dominated by relatively high and slightly variable energy conditions. This was attributed to the lack of bioturbation, silt grains intermingling with the pellets, and current conditions as indicated by the slight imbrication of the pellets. It was argued that the production of fecal pellets by deposit feeders cannot explain the deposition of mud, and instead only leads to the redeposition and rearrangement of the mud in current-bedded sediment. Instead, the pelleted sands were considered to result from the transport of pelleted mud from areas of suspension deposition into areas of more regular current deposition, and to represent the recycling of mud that was originally deposited by suspension feeders as their fecal pellets, as described by Pryor [18]. Pelleted laminations accumulated in the box cores extracted from tidal flat sediments of the Kundalika Estuary, offer renewed insights into several such geological records. The polychaete pellets deposited as mounds and strings on the surface of the tidal flats get splayed across the sediment surface as the water level gently rises through the high tide cycle. It is emphasized here that the coarse sand sized pellet aggregates seem to obliterate or overshadow the presence of associated burrows due to their concentration and/or their cementation in no particular alignment. This modern analogue could explain the formation and preservation of pelleted mud laminations in the Oligocene Vejle Fjord Formation of the Danish Tertiary. The fossilization of such fecal pellet accumulations seems possible only in the event of them being subjected to early cementation. In the Kundalika Estuary, the compact and cohesive fecal pellets are bound together in stacks by organic matter and salt matrix deposited by interstitial waters, facilitating the retention of the original shape of the pellets at this stage of early cementation. These modern and fossil occurrences of pellet beds testify the preservation potential of polychaete pellet sands in the geological record.

Another important sedimentological connotation to be emphasized is the modification of sediment texture due to the aggregation of clay and silt sized particles into sand sized sediments, thereby improving the porosity of the sediment, leading to enhanced aeration in the surficial sediments.

### Paleoenvironmental implications

The formation of pellet sands has biogeochemical implications. The alteration of sediment textures leads to better oxygenation of tidal mudflats, greatly reducing the preservation of organic matter. Interstitial oxygen in tropical, estuarine, tidal mudflats is crucial in the preservation of organic matter and preservation of peat. In this context the depth of bioturbation by the polychaetes is significant. Polychaete burrows have been reported up to a depth of 50 cm in sandy intertidal zones [7] where interstitial aeration is better. In the present study, where the substrate comprises 90 to 98% mud, the polychaete burrows are limited to a maximum depth of 20 cm within the sediment column, suggesting severe depletion in interstitial oxygen. The light halo consistently associated with these burrows represents an oxidized zone resulting from polychaete respiration. Kędzierski et al. [19] have reported light haloes lining only those *Trichichnus* burrows which are pyritized. They attribute the halo to the oxidation associated with pyritization. The complexity and distribution of the traces of the deposit feeding polychaetes discussed above suggests a developmental comparison with other composite traces, which attain different morphologies with progressive stages of development (e.g. *Hillichnus lobosensis* [20–21]). Thus, it is proposed that the making and distribution of these polychaete traces need to be revisited. Either this trace has in the past been observed in parts due to limitations in preservation, observations and/or due to ignorance about apparently inconspicuous association of its different elements.

## Conclusions

Fecal pellets and associated burrows observed in the study area can be considered as modern analogues of different the ichnogenera, namely *Alcyonidiopsis*, *Planolites*, *Coprulus*, *Tomaculum* and *Trichichnus*. Here they are reported as complex traces [22] because the pellets occur as aggregates, strings, beds, and also stuffed within burrows, which are branched, unlined, characterized by a halo and occasionally annulated within. They are created by a combination of the feeding and dwelling activities of polychaetes. The present neo-ichnological study confirms the interpretation of *Alcyonidiopsis / Tomaculum* as indicators of feeding behavior of polychaetes [23–24]. However, that they represent pascichnial behavior [25] could not be ascertained due to lack of enough evidence.
